# Developing a framework for the ethical design and conduct of pragmatic trials in healthcare: a mixed methods research protocol

**DOI:** 10.1186/s13063-018-2895-x

**Published:** 2018-09-27

**Authors:** Monica Taljaard, Charles Weijer, Jeremy M. Grimshaw, Adnan Ali, Jamie C. Brehaut, Marion K. Campbell, Kelly Carroll, Sarah Edwards, Sandra Eldridge, Christopher B. Forrest, Bruno Giraudeau, Cory E. Goldstein, Ian D. Graham, Karla Hemming, Spencer Phillips Hey, Austin R. Horn, Vipul Jairath, Terry P. Klassen, Alex John London, Susan Marlin, John C. Marshall, Lauralyn McIntyre, Joanne E. McKenzie, Stuart G. Nicholls, P. Alison Paprica, Merrick Zwarenstein, Dean A. Fergusson

**Affiliations:** 1 Clinical Epidemiology Program, Ottawa Hospital Research Institute (OHRI), The Ottawa Hospital, Civic Campus, 1053 Carling Avenue, Ottawa, ON K1Y 4E9 Canada; 2 School of Epidemiology and Public Health, University of Ottawa, Ottawa, ON Canada; 3 Rotman Institute of Philosophy, Western University, 1151 Richmond Street, London, ON N6A 5B7 Canada; 4 Clinical Epidemiology Program, Ottawa Hospital Research Institute (OHRI), The Ottawa Hospital, General Campus, 501 Smyth Road, Ottawa, ON K1H 8L6 Canada; 5 Department of Medicine, University of Ottawa, Ottawa, ON Canada; 6 Patient and Family Advisory Council, The Ottawa Hospital, Ottawa, ON Canada; 7 Health Services Research Unit, University of Aberdeen, Health Sciences Building, Foresterhill, Aberdeen, AB25 2ZD UK; 8 Department of Science and Technology Studies, University College London, 22 Gordon Square, King’s Cross, London, WC1H 0AW UK; 9 Centre for Primary Care and Public Health, Queen Mary University of London, 58 Turner Street, London, E1 2AB UK; 10 Applied Clinical Research Center, Children’s Hospital of Philadelphia, 2716 South Street, Philadelphia, PA 19146 USA; 11 Université de Tours, Université de Nantes, INSERM, SPHERE U1246, Tours, France; 12 INSERM CIC1415, CHRU de Tours, Tours, France; 13 Institute of Applied Health Research, University of Birmingham, Birmingham, B15 2TT UK; 14 Division of Pharmacoepidemiology and Pharmacoeconomics, Department of Medicine, Brigham and Women’s Hospital, 1620 Tremont Street, Boston, MA 02120 USA; 15 Center for Bioethics, Harvard Medical School, Boston, MA USA; 16 Division of Gastroenterology, Department of Medicine, Western University, London, ON Canada; 17 Division of Epidemiology and Biostatistics, Western University, University Hospital, 339 Windermere Road, London, ON N6A 5A5 Canada; 18 Children’s Hospital Research Institute of Manitoba, 513-715 McDermot Avenue, Winnipeg, MB R3E 3P Canada; 19 Department of Philosophy and Center for Ethics and Policy, Carnegie Mellon University, 150A Baker Hall, Pittsburgh, PA 15213-3890 USA; 20 Clinical Trials Ontario, 661 University Avenue, MaRS Centre, West Tower, Toronto, ON M5G 1M1 Canada; 21 St. Michael’s Hospital, Department of Surgery, University of Toronto, 30 Bond Street, Toronto, ON M5B 1W8 Canada; 22 Department of Medicine (Division of Critical Care), University of Ottawa, Ottawa, ON Canada; 23 School of Public Health and Preventive Medicine, Monash University, 553 St Kilda Road, Melbourne, VIC 3004 Australia; 24 Institute of Health Policy, Management and Evaluation, University of Toronto, Health Sciences Building, 155 College Street, Toronto, ON M5T 3M6 Canada; 25 Centre for Studies in Family Medicine, Department of Family Medicine Schulich School of Medicine & Dentistry Western University, 1151 Richmond Street, London, ON N6A 3K7 Canada

**Keywords:** Pragmatic randomized controlled trials, Clinical trials, Research ethics, Informed consent, Usual care interventions, Patient-centered research, Mixed methods, Ethics guidelines, Comparative effectiveness research, Large simple trials

## Abstract

**Background:**

There is a widely recognized need for more pragmatic trials that evaluate interventions in real-world settings to inform decision-making by patients, providers, and health system leaders. Increasing availability of electronic health records, centralized research ethics review, and novel trial designs, combined with support and resources from governments worldwide for patient-centered research, have created an unprecedented opportunity to advance the conduct of pragmatic trials, which can ultimately improve patient health and health system outcomes. Such trials raise ethical issues that have not yet been fully addressed, with existing literature concentrating on regulations in specific jurisdictions rather than arguments grounded in ethical principles. Proposed solutions (e.g. using different regulations in “learning healthcare systems”) are speculative with no guarantee of improvement over existing oversight procedures. Most importantly, the literature does not reflect a broad vision of protecting the core liberty and welfare interests of research participants. Novel ethical guidance is required. We have assembled a team of ethicists, trialists, methodologists, social scientists, knowledge users, and community members with the goal of developing guidance for the ethical design and conduct of pragmatic trials.

**Methods:**

Our project will combine empirical and conceptual work and a consensus development process. Empirical work will: (1) identify a comprehensive list of ethical issues through interviews with a small group of key informants (e.g. trialists, ethicists, chairs of research ethics committees); (2) document current practices by reviewing a random sample of pragmatic trials and surveying authors; (3) elicit views of chairs of research ethics committees through surveys in Canada, UK, USA, France, and Australia; and (4) elicit views and experiences of community members and health system leaders through focus groups and surveys. Conceptual work will consist of an ethical analysis of identified issues and the development of new ethical solutions, outlining principles, policy options, and rationales. The consensus development process will involve an independent expert panel to develop a final guidance document.

**Discussion:**

Planned output includes manuscripts, educational materials, and tailored guidance documents to inform and support researchers, research ethics committees, journal editors, regulators, and funders in the ethical design and conduct of pragmatic trials.

## Background

### Pragmatic versus explanatory trials

Pragmatic trials aim to determine if an intervention works in real-world settings, so that results can be generalized to everyday practice and support decision-making by patients, providers, and health system leaders; contrastingly, explanatory trials aim to determine if and how an intervention works under well-defined and highly controlled conditions [1]. While differences between explanatory and pragmatic attitudes in trials were first highlighted in a seminal article by Schwartz and Lellouch five decades ago [2], interest in pragmatic trials has increased dramatically in recent years [3]. Accordingly, reporting guidelines for pragmatic trials have been recently published [4].

In practice, trials are seldom purely pragmatic or purely explanatory, but various design choices can make a trial more or less pragmatic. The PRagmatic Explanatory Continuum Indicator Summary (PRECIS-2) was developed to help trialists identify explicit design choices that can shift a trial towards being more pragmatic [5]. PRECIS-2 has nine dimensions along which a trial can be scored from very explanatory to very pragmatic. In brief, trials that are more pragmatic have broader eligibility criteria, recruit participants at the time of presentation, include a diverse range of settings that mirror real-world circumstances, do not require highly specialized training or research personnel, give healthcare providers flexibility in how the intervention is delivered, require no special strategy for monitoring protocol compliance, follow and monitor patients as in routine clinical practice, have clinically meaningful and patient-centered outcomes, and include all randomized patients in analysis.

Explanatory trials, more often conducted on innovative medical products and devices for regulatory purposes, usually do not fully explicate the benefits and harms of interventions relative to existing alternatives and hence do not address the central question of what is likely the best (among the available) options for particular patients. In contrast, pragmatic trials test a much wider range of interventions, including diagnostic, preventive, therapeutic, and delivery system interventions. They may test new interventions against current routine interventions or the comparative effectiveness of different routine interventions head-to-head. When designed appropriately, they may address not only whether an intervention works, but more importantly, for whom and under what conditions. They may test different quality and service improvement interventions as well as knowledge translation interventions. Pragmatic trials therefore offer an important opportunity to improve patient health and health system outcomes by reducing variations in care, improving uptake of evidence-based practice, and reducing costs. For these reasons, the need for more pragmatic trials has been identified as a priority by governments worldwide [6–10].

This manuscript presents the study protocol for a four-year, interdisciplinary, mixed methods research project with the ultimate goal to develop internationally accepted guidance for the ethical design and conduct of pragmatic trials. Although there are many innovative observational study designs which can be used to evaluate interventions and produce new knowledge that informs decision-making [11], here we focus exclusively on intervention studies that use randomization. We allow for a broad range of intervention types including diagnostic, preventive, therapeutic, knowledge translation, and delivery system interventions. See Table 1 for a brief glossary of terms used in this manuscript.

**Table 1:** Glossary of terms

Term	Definition assumed in this manuscript
Pragmatic trial	A trial whose purpose is to evaluate the effectiveness of an intervention with the view to informing a decision about a healthcare policy or practice; key characteristics are broad eligibility criteria and patient-centered outcomes to maximize generalizability and applicability.
Intervention	Includes diagnostic, preventive, therapeutic, and delivery system interventions.
Randomized controlled trial	A research study in which, using a random mechanism, human participants are prospectively assigned (whether as individuals or in groups) to one or more interventions (which may include usual care or other competing interventions), to evaluate the effects of those interventions on health-related biomedical or behavioral outcomes.
Usual care interventions	Treatments or procedures that have been accepted by medical experts as appropriate treatments or procedures for a given type of disease or condition and are commonly used by healthcare professionals.
Knowledge translation interventions	An intervention designed to improve the uptake of research evidence in practice and reducing barriers and facilitators inherent in this process.
Gatekeepers	Individuals or bodies that represent the interests of community members, communities, or organizations participating in pragmatic trials.

### Ethical issues in pragmatic trials

Trials undertaken in real-world settings raise substantial ethical issues that have not yet been fully addressed [12–15]. These ethical issues arise not only from the push towards a greater degree of pragmatism (e.g. along the nine PRECIS-2 dimensions), but are closely tied to the types of interventions, as well as the choice of study design. In addition to established study designs which use patient randomization (see Example 1 below), pragmatic trials include some emerging designs and approaches which capitalize on methodological and statistical innovations as well as the availability of registries and routinely collected health data (e.g. cohort multiple randomized controlled designs [16], randomized registry trials [17], cluster cross-over trials [18], and stepped wedge cluster randomized trials [18, 19]). The cohort multiple randomized design and randomized registry trials are examples of pragmatic trial designs that facilitate the evaluation of usual care interventions embedded within routine settings, but their ethical implications remain unclear. Cluster randomization [20] may be used to randomize entire medical practices or hospitals to differing interventions and is a natural choice for evaluating service delivery or other health system level interventions; however, with the push towards more pragmatic trials, this design is increasingly being used to evaluate individual-level interventions (i.e. interventions that, in theory, could have been evaluated using traditional patient randomized designs). While the *Ottawa Statement on the Ethical Design and Conduct of Cluster Randomized Trials* [21] provides explicit guidance for cluster randomized trials, the use of this design in the case of usual care individual-level interventions raises additional ethical issues (see Example 2 below); moreover, the stepped wedge cluster randomized design raises its own unique ethical issues (Example 3).

Table 2 describes a preliminary framework of nine ethical issues; the pragmatic, intervention, and design characteristics that give rise to these issues; and their potential implications for researchers and research ethics committees. This framework was developed based on an initial scoping review [12] and will be further developed and refined during our project.

**Table 2:** Preliminary framework of ethical issues raised by pragmatic randomized controlled trials (pragmatic RCTs)

	Ethical issue	Characteristics of pragmatic RCTs raising ethical issue	Potential ethical issues for researchers and research ethics committees
1.	Are activities in pragmatic RCTs research or practice?	• Commonly evaluate interventions used in routine clinical practice • Seek to evaluate interventions and assess outcomes in usual care settings • Favor unobtrusive data collection via routinely collected sources	• Difficult to clearly separate research from clinical practice • Advocates of *learning health systems* have challenged the research-practice distinction, but unclear if a new system will be preferable and how it satisfies current research regulations
2.	What level of oversight is required for pragmatic RCTs?	• May involve usual care interventions or interventions posing no more than minimal risk • Address questions that directly inform decision-making by patients and healthcare providers • May have quality and service improvement as a central goal	• Current oversight procedures are time-consuming, costly, and overly complex • Some advocate that low-risk pragmatic RCTs do not require more stringent oversight than clinical practice as all patients receive an intervention used in routine clinical practice
3.	Which study designs are appropriate in pragmatic RCTs?	• Aim to maximize representativeness; may require larger sample sizes to have adequate power • May favor novel designs, including cluster randomized, stepped wedge, registry, and cohort multiple designs	• Study designs can have differing implications for trial feasibility and logistics which must be balanced against internal and external validity • RCTs can substantially simplify trial logistics and facilitate recruitment, but ethical justification unclear • Interventions of unknown effectiveness may be rolled out to all clusters in stepped wedge trials
4.	Who are the research participants in pragmatic RCTs?	• May involve stakeholders at multiple levels: health system; hospital; provider; patient • May evaluate interventions targeting one group, but measure outcomes on another	• Identification of research participants influences the scope of research ethics review, benefit–harm analysis, and informed consent procedures
5.	Do patients and providers have an ethical obligation to participate in pragmatic RCTs?	• Seek to provide highly relevant evidence for patients, providers, and health systems • High degree of flexibility in intervention delivery and data collection implies low burden of study participation	• Unclear if health system leaders and providers have a prima facie ethical duty to seek to continually improve the delivery and outcomes of healthcare • Patients receive benefits from the health system which may engender an obligation to participate in research
6.	From whom, how, and when is informed consent required in pragmatic RCTs?	• May involve usual care interventions or interventions posing no more than minimum risk • May use solely routinely collected data for outcome assessment • Informed consent can pose barriers to representative recruitment, be a burden on staff, add logistical complexity, and be costly • May expose differing groups of participants to different aspects of the research • May use cluster-level interventions that are difficult or impossible to avoid	• Unclear if simplified (“altered”) or no consent procedures are acceptable and under which conditions • Unclear what aspects of research must be disclosed to research participants • Unclear if informed consent is needed from health system leaders, decision-makers, health providers
7.	Who are the gatekeepers in pragmatic RCTs and what are their responsibilities?	• May have an impact on group or institutional interests • May involve a variety of gatekeepers, including ministry of health, hospital administrator, and data custodians	• Lack of clarity regarding the role of gatekeepers, whose interests they protect and scope of their authority • Unclear when community consultation is appropriate
8.	How should harm–benefit analyses be conducted in pragmatic RCTs?	• Procedures administered by a wide range of providers under imperfect conditions and in a variety of institutional settings • May evaluate policy or health delivery system interventions or usual care interventions • May use delayed implementation of intervention (stepped wedge design)	• Unclear how to determine the boundaries of appropriate provider experience and training • What constitutes usual care • Benefit–harm analysis in studies of policy or healthcare delivery is unclear • Unclear when it is appropriate to delay delivery of interventions in stepped wedge designs
9.	How ought vulnerable groups be protected in pragmatic RCTs?	• Seek to study a representative group including individuals traditionally considered vulnerable (lower socioeconomic strata, children, pregnant women, prisoners) • Health providers/ employees may be exposed to social risks, including reputational or professional harm	• Some criticize the traditional approach to defining vulnerability and have proposed alternative definitions • Presence of vulnerable participants may be hidden • Unclear how to protect employees in pragmatic RCTs • Unclear who should set the research agenda for pragmatic RCTs

*RCT* randomized controlled trial, *CRT* cluster randomized trial

### Examples of pragmatic randomized controlled trials

For illustrative purposes, we present three examples of pragmatic trials that raise important ethical issues needing guidance.

#### Example 1: patient-randomized trial comparing usual care interventions (SUPPORT)

The SUPPORT trial [22] sought to determine the optimal level of supplemental oxygen on incidence of retinopathy of prematurity and mortality in preterm infants. Infants were randomized to high and low oxygen saturations, both of which fell within the range used routinely in practice. Lower oxygen levels were found to reduce retinopathy but increase mortality, leading the authors to urge caution in their use. In response to an anonymous complaint by parents, the US Office for Human Research Protections launched an investigation into the trial [23]. It determined that investigators failed to adequately inform parents of “reasonably foreseeable risks” as consent materials did not list visual impairment and death as research risks. This incited considerable debate in the literature [24–27] centered almost exclusively on considerations of risk and risk communication and reflecting a preoccupation with US regulations. A central ethical issue raised by the SUPPORT trial is: *Should usual care interventions be considered part of research or clinical practice*? Other issues raised are: *What constitutes usual care*? *Can a study be considered “minimal risk” if its endpoints include serious impairments such as blindness or death? Do parents need to be informed that their child is participating in a trial? At what level of detail do the study interventions need to be disclosed to participants? What potential benefits and harms ought to be disclosed? What type of research ethics review is appropriate*?

#### Example 2: cluster randomized registry trial comparing usual care individual-level interventions implemented as policy interventions at the cluster-level (FLUID)

The FLUID trial is a pragmatic cluster randomized cross-over trial comparing resuscitation with Ringer’s Lactate versus normal saline on death and hospital readmissions in hospitalized patients, with outcomes assessed using health administrative data [28]. Both fluids are considered usual care interventions that have been available for decades and are administered to many hospitalized patients. While fluid administration is an individual-level intervention, the study becomes feasible only when interventions are implemented as hospital policies (i.e. at the cluster level) with a waiver of patient informed consent. In particular, it is essential to have only one type of study fluid available throughout the hospital to minimize the risk of contamination due to a patient receiving both types of study fluids in different areas of the hospital. It would also be logistically challenging and very costly to recruit and randomize individual patients within all areas of participating hospitals. In contrast, the availability of routinely collected data available for all patients permits the conduct of a very cost-efficient trial involving hospitals across the province, without the need to recruit patients for data collection. However, the FLUID trial raises several ethical issues: *What is an appropriate justification for adopting cluster randomization? When individual-level interventions are implemented institution-wide as a policy in a cluster randomized trial, may one proceed without patient consent? Do patients need to be notified about the trial; if so, how? Is consent required for the use of routinely collected participant data?*

#### Example 3: stepped wedge trial of a quality improvement intervention (Surgical Checklist Trial)

The Surgical Checklist Trial was a stepped wedge trial randomizing surgical units in two hospitals in Norway to evaluate the impact of the World Health Organization Surgical Safety Checklist on morbidity, mortality, and length of hospital stay [29]. The stepped wedge design is characterized by the fact that clusters (here, surgical units) cross gradually and in random sequence from the control to the intervention condition, with all clusters exposed to the intervention by the end of the trial. As stated by the investigators, the 19-item checklist consists of an oral confirmation by surgical teams of the completion of the basic steps for ensuring safe delivery of anesthesia, prophylaxis against infection, effective teamwork, and other essential practices in surgery. Compliance with the checklist was assessed prospectively by nurses. All patient outcomes were collected from hospital administrative databases. The regional research ethics committee advised the investigators that the study was considered clinical service improvement and that research ethics approval and patient informed consent were not required. The Surgical Checklist Trial raises several ethical issues: *Should trials evaluating quality and service improvement be considered research? What are the ethical implications if health system leaders conduct (potentially less robust) quality improvement studies rather than rigorous pragmatic randomized controlled trials or refrain from conducting important studies due to perceived ethical barriers? Should health professionals targeted by study interventions be considered research participants? If study participation poses no more than minimal risk, is patient or provider consent required? What is the harm–benefit balance of exposing all surgical units to the intervention by the end of the study?*

### Challenges to the conventional framework for research ethics

As illustrated by our three examples, pragmatic trials raise important ethical issues that have not yet been satisfactorily addressed. Existing ethical and regulatory frameworks were developed primarily for trials with explanatory aims, i.e. focusing on efficacy and safety of experimental interventions for marketing approval. With the move towards the conduct of more pragmatic trials, existing ethics guidance is becoming more difficult to interpret and apply and may not be sufficient to address these new ethical issues. On the one hand, the absence of clear guidance may put trials at risk of being accused of exposing participants to inadequate protections (real or perceived) (e.g. the SUPPORT trial [23]). It is essential to avoid undermining the trust that patient communities have in the research enterprise. On the other hand, strict enforcement of conventional rules is likely to pose unnecessary obstacles, undermine scientific quality or impede improvements in patient health and health system outcomes. Unless these ethical issues are addressed, important research with large potential healthcare benefits may be impeded or may go ahead without adequate safeguards.

### Objectives

The overarching goal of our project is to develop guidance for the ethical design and conduct of pragmatic trials. Specific objectives are to:

1. Systematically identify ethical issues arising from pragmatic trials;
2. Document ethical practices in completed and ongoing pragmatic trials;
3. Elicit views and experiences of trialists, methodologists, chairs of research ethics committees, trial participants, and health system leaders;
4. Develop novel ethical solutions informed by ethical analyses;
5. Generate ethics guidance through a consensus process with an independent expert panel;
6. Disseminate tailored guidance to stakeholders.

## Methods

### Overview

**Fig. 1:**
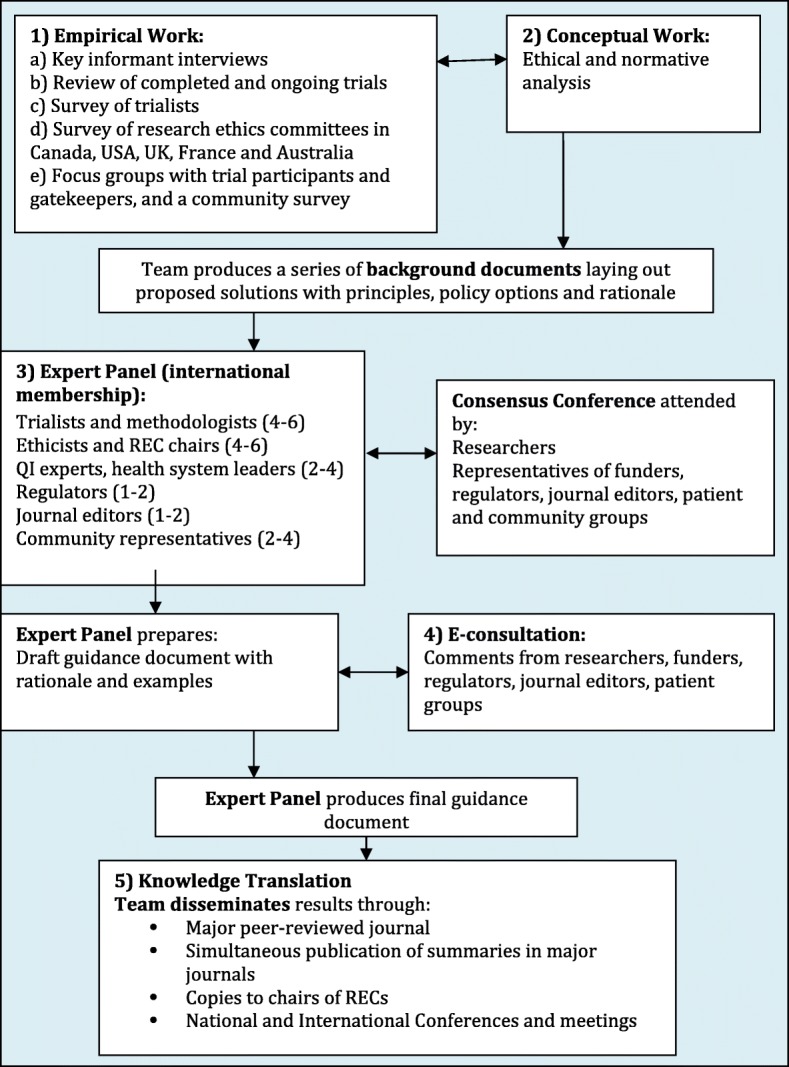
Overview of project phases

Our project, summarized in Fig. 1, consists of both empirical and conceptual work and concludes with a consensus development process and knowledge translation. The empirical work, to be completed over the first three years, consists of five studies:

1. Key informant interviews with a small group of 24–40 experts;
2. Identification and review of a random sample of 300 completed and ongoing pragmatic trials;
3. Survey of trialists (investigators of the 300 pragmatic trials);
4. Survey of a random sample of chairs of research ethics committees in Canada, USA, UK, France, and Australia; and
5. Embedded focus group discussions and a community survey with trial participants (e.g. patients) and gatekeepers (i.e. those who have ability to allow or deny access to trial participants).

All outputs will inform the ethical analysis in the conceptual work.

The conceptual work will be an ethical analysis of the identified ethical issues from the empirical work, resulting in a series of publications outlining proposed solutions with principles, policy options, and rationales. The empirical and conceptual work will proceed concurrently to allow the empirical and ethical analyses to be mutually informative.

The consensus process, occurring upon completion of the empirical and conceptual work, will involve identifying an international expert panel of trialists, ethicists, chairs of research ethics committees, regulators, funders, and community representatives and organizing a consensus conference attended by the panel, as well as invited researchers, representatives of funding agencies, regulators, journal editors, and patient and community groups. In addition, we will engage in e-consultation with the broader research community, funders, regulators, journal editors, and patient groups. The panel will produce the final ethics guidance document which will be disseminated to stakeholders by the research team.

### Empirical studies

#### Study 1: key informant interviews

The objective of this study is to conduct interviews with a small group of pragmatic trial experts and stakeholders (trialists, ethicists, methodologists, chairs of research ethics committees, health system leaders, quality improvement experts, and patient representatives on research study teams) to generate a thorough understanding of types of ethical issues arising in the practice of pragmatic trials from a variety of perspectives. Informants will be selected using a purposive sampling strategy, augmented through snowball sampling. Potential interviewees will be selected across a broad range of jurisdictions and clinical areas to reflect a range of experiences, including lower- and middle-income countries. The sample size will be determined to ensure representation from all targeted stakeholders and by when saturation is reached (i.e. when new interviews cease to provide fresh information) [30–34]. Based on our prior experience with similar studies [35], we anticipate that 24–40 interviews will be required. Interviews are expected to last 1 h and will be audio recorded. A semi-structured interview guide will allow participants to respond freely, to illustrate concepts, and to present perspectives that the interviewer can probe further. To monitor the progress of the interviews and permit follow-up of issues that may emerge from the data, interviewing, transcription, and analysis will proceed concurrently. The interview guide may evolve as a typology of ethical issues begins to emerge. Recordings will be transcribed and verified before analysis. Data will be imported into a qualitative software package (NVivo 11) to facilitate thematic coding, evaluation, and analysis. The results will be used to formulate a typology of ethical issues arising from pragmatic trials, to be addressed in the conceptual work. It will also inform data extraction and questionnaire items for studies 2–5.

#### Study 2: review of published trials

The objectives of this study are to select and review a random sample of recently completed and ongoing trials that have more pragmatic (than explanatory) aims, to describe ethical characteristics, identify ethical challenges reported, the circumstances under which they arise, and how they are being addressed. We anticipate challenges in identifying a sample of “pragmatic” trials given wide variation in definitions and inconsistent and unreliable reporting of trial design. We will develop objective and reproducible criteria to characterize a trial as having pragmatic aims, as well as the conditions under which those criteria apply. We will work with an information scientist to develop a sensitive and specific electronic search strategy to identify a sample of trials. To develop and validate an electronic search strategy, we will use a multi-pronged approach to identify a gold standard set of trials meeting our criteria for testing the search strategy, including: (1) pragmatic trials conducted by the investigator team and our extensive networks; (2) pragmatic trials identified in the key informant interviews; (3) databases of funded pragmatic trials in Canada, the USA, UK, France, and Australia; (4) a database of pragmatic trials maintained by the PRECIS group [36]; (5) demonstration projects by the National Institutes of Health Collaboratory [37] and PCORnet [38]; and (6) trials included as exemplars in recent publications about pragmatic trials. The proportion of these pragmatic trials retrieved by the search strategy will be calculated and used to refine the search strategy if necessary as done in our previous work [39]. Once validated, the search strategy will be used to select a random sample of 300 pragmatic trials. As the ethical landscape may have been changing in recent years, we will include completed trials as well as study protocols for ongoing trials. Eligibility criteria, to be refined, will include trials or study protocols published in the past five years by investigators in Canada, UK, USA, France, and Australia, including trials conducted in lower- and middle-income countries. Items for extraction will be generated based on team discussion and key informant interviews. After pilot testing, two reviewers will independently extract data from each trial report. Discrepancies between reviewers will be identified and resolved by discussion with a third reviewer if required. Prevalence of ethical issues and practices arising from pragmatic design features, study design, and type of study interventions will be described overall and within subgroups of interest where feasible (e.g. over time, between countries, study sponsors). Methodological and reporting quality of the included trials will be collected and evaluated against major methodological and reporting criteria as done in our previous work [40, 41]. The preliminary ethics framework will be updated as extractions proceed.

#### Study 3: survey of trialists

The objective of this study is to gather more detailed information about practices and experiences identified in our review of pragmatic trials. After pilot testing, we will administer a survey consisting of open- and closed-ended items to corresponding authors of the sample of 300 trials. The primary mode of survey administration will be web-based, but alternatives (paper, telephone) will be considered to increase the response rate. A series of contacts (pre-notifications, notifications, and reminders) based on Dillman’s recommendations for the implementation of mail and Internet surveys will be used [42]. The survey will be used to characterize the ethical conduct, review, and reporting of pragmatic trials from the perspectives of trialists. We will offer respondents a $30 gift certificate or donation on behalf of the respondent in appreciation for their time. We will use questionnaire personalization—a previously published methodology developed by our team—to gather more detailed information about aspects of the published trial [43]. The anticipated response rate, based on previous experience with this population and methodology, is 65% [44]. Results from the survey will be compared to results from the published trial to assess adequacy of reporting of ethical issues and to describe implications of design choices (e.g. impact of alternative consent models on study recruitment and risks of selection bias). Potential non-response bias will be assessed by comparing characteristics of respondents and non-respondents using information in trial reports. We will describe the use of gatekeepers (i.e. individuals or bodies that represent the interests of community members, communities, or organizations [45]), use of consent waivers or alternative consent models (e.g. “streamlined consent” [12]), and details about information conveyed to participants in each study arm. We will describe the type of ethics review required, perceived impact on the timing of implementation, ethical and scientific quality of the trial, and uniformity of process and decisions in multicenter trials. We will explore the possibility of requesting informed consent documents and research ethics application forms and protocols for a subset of trials. Results will inform the ethics framework for analysis in the conceptual work.

#### Study 4: survey of research ethics committee chairs

The objective of this study is to gather information on the views, practices, and experiences of research ethics committee chairs in Canada, the USA, UK, France, and Australia. We will aim to select all research ethics committees that review clinical trials in Canada (approximately 200), the UK (approximately 100), Australia [46] (approximately 200), France (approximately 39) [47], and a random sample of 200 from over 9000 Institutional Review Board Organizations in the USA [48]. We chose these five countries primarily based on logistical considerations: our team members have connections with research ethics organizations in these countries which will help facilitate participation. Canada does not maintain a list of research ethics committees. We will use a strategy previously developed by our team to identify eligible committees [49]. It involves integrating internet searches with a list of Institutional Review Board Organizations maintained by the US Office of Human Research Protections. Given that biomedical and non-biomedical Institutional Review Boards are not differentiated in this list, we will use stratification by National Institutes of Health funding levels to increase the efficiency of identifying Boards with relevant experience reviewing clinical research.

Questionnaire items will be informed by the preliminary ethics framework, key informant interviews, and results from the trialist survey. Questionnaires will consist of open- and closed-ended items and include a series of scenarios. After pilot testing, a series of contacts (notifications and reminders) based on Dillman’s recommendations for Internet surveys will be used [42]. We will likely encounter challenges in ensuring an adequate response rate; based on previous experience with surveying this population, we expect a response rate of approximately 35% [49]. We will deliberately keep questionnaires short and adhere to recent recommendations for improving response rates [42]. Results will be summarized using descriptive statistics and compared across subgroups (e.g. country, size and type of committee, years of experience). Where feasible, potential non-response bias will be assessed by comparing characteristics of respondents and non-respondents using information on research ethics committee websites. Questionnaires will be prepared in both English and French. Open-ended responses will be analyzed thematically. Results will inform the ethics framework for analysis in the conceptual work and knowledge translation activities to research ethics committees.

#### Study 5: focus group discussions and community survey

The objective of this study is to gather information on the views and experiences of trial participants or prospective trial participants (e.g. patients), gatekeepers (organizational leadership, medical directors), and communities. Our research team members are involved in 15–20 pragmatic trials at any one time. We will identify one ongoing or recently completed pragmatic trial in each country, ensuring a range of types of pragmatic trials. We will conduct focus groups with eligible trial participants (with permission from the responsible research ethics committees and the chief investigator). We anticipate five focus groups with patients and five with gatekeepers. Focus groups will be 1–2 h in length and involve six participants per group. A semi-structured discussion guide will be used to gather information on participants’ experiences with the trial including recruitment, informed consent, perceived benefits and harms, any privacy concerns, and satisfaction with the trial. Among eligible prospective participants, we will explore potential reasons for non-participation. Discussions will be recorded, transcribed verbatim, and verified by the facilitator before analysis in NVivo 11 [50]. To monitor progress and permit follow-up of issues that may emerge from the data, discussions, transcription, and analysis will proceed concurrently [51]. For focus groups not conducted in French, interview guides will be developed in English by the study team and then translated into French by bilingual members of the research team. To verify the accuracy of the translation, all guides will be independently back-translated. All focus group discussions will be transcribed in the language in which the group was conducted. Non-English transcripts will be translated into English and then independently back-translated to the original language. A Canadian pragmatic trial will be used to design a survey of community members targeted by the trial (e.g. diabetes patients, hospital patients). Quantitative and qualitative analyses will be used to summarize results from the community survey. Results will be used to inform the ethical analysis in the conceptual work.

### Ethical analysis

The ethical analysis will be an intensive process run concurrently with the empirical work and will extend over a period of three years. Conceptual work in bioethics is not amenable to the degree of a priori methodological specification that is expected of empirical research. Reproducibility is an indispensable feature of rigorous science, necessitating the clear statement of hypotheses and experimental methods upfront. Rigorous conceptual work in ethics begins with the articulation of clear and important questions and is realized in the construction of careful and clear analysis of the relevant concepts and of ethical arguments in peer-reviewed publications and policy reports [52]. The ethical analysis in this project will be based on an evolving framework of ethical issues developed using results from an extensive literature search conducted in preparation for this proposal (see Table 2) and revised using results from the five empirical studies. For each identified set of core ethical issues, an in-depth and written ethical analysis will be prepared. An extensive review of the scholarly literature will document and critically analyze arguments proffered for and against ethical positions. The ethical analysis will seek to synthesize foundational documents, regulations, and arguments in the literature into a coherent solution. Where disagreement among the various sources cannot be resolved by critical analysis, the contours of the ethical dispute will be documented. The ethical analysis will result in a series of background documents laying out proposed solutions with principles, policy options, and rationale and will also be submitted for peer-reviewed publication. These documents will be used as background materials in the consensus process.

### Expert panel and consensus process

An international expert panel will be convened to develop ethics guidance. The composition of the panel is expected to be 4–6 pragmatic trialists and methodologists, 4–6 ethicists and chairs of research ethics committees, 2–4 quality improvement experts and health system leaders, 1–2 regulators, 1–2 journal editors, and 2–4 community members. No more than one-third of the members of the panel will be drawn from the research team and adequate representation from a broad range of countries including lower- and middle-income countries will be sought. The panel will be provided with output from the empirical studies as well as the documents prepared during the ethical analysis one month in advance of a three-day consensus conference which will also be attended by invited researchers and representatives from major funding bodies, regulators, journal editors, and community groups. The conference will consist of both open and closed sessions. At the open sessions, proposed ethics guidance with supporting ethical analysis will be presented by the research team and comments and discussion invited from attendees. The panel will then meet in closed sessions to discuss and formulate draft guidance. Based on previous experience with the consensus process [21], we anticipate that at the beginning of the meeting, the panel will set rules for debate, handling of disagreements, and how to achieve consensus. We do not expect to use a majority voting system but anticipate that agreement will be reached through discussion, with documentation of disagreements where they exist. After the meeting, a draft guidance document will be produced by a writing committee and refined after further discussion with the panel. An e-consultation process will be launched to invite comments from the broader research community, funders, regulators, journal editors, and community groups. Based on results from this process, the writing committee will make revisions and produce the final consensus guidance.

## Discussion

The goal of our international, interdisciplinary collaboration is to develop, publish, and promote the uptake of guidance for the ethical design and conduct of pragmatic randomized controlled trials. We seek to create a novel approach to the ethics of pragmatic trials that improves upon the existing literature, which has been criticized for lacking convincing arguments grounded in ethical principles [53], including claims based on erroneous assumptions [54], appealing mainly to regulations in particular jurisdictions [55–57], offering speculative solutions with no guarantee of improvement over existing oversight procedures [58], and failing to reflect a broad vision of protecting the liberty and welfare interests of research participants [59]. The proposed process is informed and enriched by our previous experience with developing ethics guidance for cluster randomized trials [21, 60]. The planned output includes manuscripts, educational materials, and tailored guidance documents. Our proposed project is novel in that it: (1) involves close collaboration between clinical trialists, ethicists, and methodologists; (2) combines concurrent empirical and ethical analysis in a mutually informative approach; (3) integrates views and experiences of stakeholders (e.g. trialists, chairs of research ethics committees, health system leaders, community members); and (4) aims to generate guidance rooted in internationally accepted ethical principles rather than regulation specific to one jurisdiction. We expect that the study outputs will be of interest to a wide range of knowledge users including trialists, healthcare professionals, ethicists, research ethics committees, journal editors, regulators, health system leaders, research funders, and patient groups. Guidance will facilitate the conduct of research important to patients, clinicians, and the healthcare system, while upholding the highest ethical standards in research. While the scope of the planned guidance is intended to be international, some of the empirical studies (e.g. focus groups) will be geographically restricted based on logistical and feasibility considerations.

Our knowledge translation strategy will be guided by the Canadian Institutes of Health Research Guide to Knowledge Translation Planning [61]. We will use our considerable informal networks nationally and internationally to disseminate our findings. Work stemming from the research will be submitted for presentation at national and international conferences and meetings targeting specific stakeholder groups (e.g. journal editors, funders, regulators). The final ethics guidance document will be published in a major journal with summaries published simultaneously in other major journals. Educational material for researchers and research ethics committees will be developed. Social media will be used to communicate results to the public.

## Abbreviations

CRT: Cluster randomized trial
PRECIS – 2: PRagmatic Explanatory Continuum Indicator Summary
QI: Quality improvement
RCT: Randomized controlled trial
REC: Research Ethics Committee
